# Correlation Between Vitamin D Deficiency (25(OH)D3) and the Severity of Purulent Oropharyngeal Infections

**DOI:** 10.3390/jcm14072410

**Published:** 2025-04-01

**Authors:** Florian Ciprian Venter, Timea Claudia Ghitea, Adrian Nicolae Venter, Amin-Florin El-kharoubi, Mousa El-kharoubi, Evelin Claudia Ghitea, Marc Cristian Ghitea, Amina Venter

**Affiliations:** 1 Doctoral School of Biological and Biomedical Sciences, University of Oradea, 410087 Oradea, Romania; cypry_85@yahoo.com (F.C.V.); aminaadnan2005@yahoo.com (A.V.); 2 Pharmacy Department, Faculty of Medicine and Pharmacy, University of Oradea, 410068 Oradea, Romania; 3 Bihor Clinical County Emergency Hospital, 410169 Oradea, Romania; venteradrian2017@gmail.com (A.N.V.); amin_kharubi@yahoo.com (A.-F.E.-k.); 4 The County Emergency Clinical Hospital of Târgu Mureș, 540136 Târgu Mureș, Romania; elkharoubimousa@gmail.com; 5 Faculty of Medicine and Pharmacy, University of Oradea, 410068 Oradea, Romania; ghitea.evelinclaudia@gmail.com (E.C.G.); ghitea.marc@gmail.com (M.C.G.)

**Keywords:** vitamin D, oropharyngeal infections, peritonsillar phlegmon, cervical abscess, severity of infection, length of hospitalization, surgery, inflammatory markers, immunity

## Abstract

**Background:** Vitamin D plays a crucial role in immune system function, and its deficiency has been associated with an increased risk of infections. This study investigates the relationship between vitamin D deficiency and the severity of purulent oropharyngeal infections, considering the need for surgical interventions and the duration of hospitalization. **Materials and Methods:** This retrospective study included patients diagnosed with peritonsillar phlegmons, laterocervical abscesses, and peritonsillar abscesses. Patients were categorized based on their vitamin D levels: deficiency (<30 ng/mL) and optimal levels (≥30 ng/mL). The clinical parameters, length of hospitalization, and type of treatment were analyzed. Statistical analyses included Student’s *t*-test, the chi-square test, and ANOVA to assess differences between groups. **Results:** Patients with vitamin D (25(OH)D3) deficiency had a significantly longer hospital stay (8.50 days vs. 3.24 days, *p* = 0.001) and required more frequent surgical interventions (55.6% vs. 27.8%, *p* = 0.002) compared to those with optimal vitamin D levels. A trend toward more complex treatment regimens was also observed, although this relationship was not statistically significant (*p* > 0.05). **Conclusions:** These findings suggest that vitamin D (25(OH)D3) deficiency may contribute to a more severe course of oropharyngeal infections, increasing the need for invasive treatments and prolonging hospitalization. This highlights the importance of monitoring vitamin D (25(OH)D3) levels and the potential benefits of supplementation in preventing and managing severe upper respiratory tract infections.

## 1. Introduction

Purulent oropharyngeal infections, such as peritonsillar phlegmons and cervical abscesses, present with varying degrees of severity, sometimes requiring surgical interventions and complex treatments [1]. The progression of these infections is influenced by multiple factors, including immunological status, associated comorbidities, and the patient’s nutritional balance. In recent years, vitamin D has gained attention as a potential factor involved in immune response regulation and susceptibility to severe infections [2,3].

Vitamin D plays a crucial role in modulating the immune system, exerting immunomodulatory effects on both innate and adaptive immunity [4]. Studies have shown that vitamin D deficiency is associated with an increased risk of respiratory infections and greater severity of bacterial infections [5,6,7]. The mechanisms through which vitamin D influences immune responses include stimulating the production of antimicrobial peptides, reducing excessive inflammation, and modulating macrophage and T-lymphocyte activity [8,9].

Given these effects, it has been suggested that patients with vitamin D deficiency may be at higher risk of developing severe oropharyngeal infections [10]. This hypothesis warrants further investigation to determine whether there is a correlation between serum vitamin D levels and the severity of purulent oropharyngeal infections, particularly regarding the need for surgical intervention and the duration of hospitalization [11,12,13]. The present study aims to explore this relationship, providing new insights into the role of vitamin D in managing patients with severe upper respiratory tract infections.

The objective of this study is to evaluate the impact of vitamin D deficiency (as a precursor to 25(OH)D3) on the severity of purulent oropharyngeal infections by assessing relevant clinical parameters such as the need for surgical intervention and the length of hospital stay. However, we acknowledge that the retrospective nature of this study limits our ability to establish causality. 

While our study identifies significant associations, it cannot definitively prove that vitamin D deficiency causes more severe infections. Future prospective studies are needed to establish a causal relationship between vitamin D levels and infection severity. Through this research, we seek to highlight the potential role of vitamin D as both a prognostic marker and a possible preventive factor in the management of severe upper respiratory tract infections.

## 2. Materials and Methods

### 2.1. Study Design

This retrospective observational study was conducted on a sample of patients diagnosed with severe oropharyngeal infections in a specialized medical center. The study analyzed patient records collected between January 2019 and December 2023. The sample size reflects the application of strict inclusion criteria and the patient population of a specialized referral center. While the sample size in this study was adequate for identifying initial associations, it is relatively small and limited to a specific geographic region. A larger, more diverse sample, including patients from multiple centers and regions, would help improve the generalizability of the results. Future studies should aim to include a broader population to better reflect the variability in vitamin D levels and infection outcomes across different geographic areas. Patient data were collected from medical records and included demographic information, serum vitamin D (25(OH)D3) levels, and infection severity.

### 2.2. Inclusion and Exclusion Criteria

#### 2.2.1. Inclusion Criteria

Patients diagnosed with peritonsillar phlegmons, laterocervical abscesses, or peritonsillar abscesses.

Availability of serum vitamin D (25(OH)D3) levels at the time of diagnosis, (JusChek, Bucharest, Romania).

Complete medical data regarding the infection severity, length of hospitalization, and need for surgery.

#### 2.2.2. Exclusion Criteria

Patients with autoimmune diseases or chronic conditions affecting vitamin D metabolism.

Recent therapy with vitamin D (25(OH)D3) supplements that could influence the results.

Incomplete or missing relevant data in medical records.

### 2.3. Description of Study Groups

Patients included in the study were categorized based on serum vitamin D (25(OH)D3) levels into three groups: deficiency (0–29.99 ng/mL), optimal level (30–99.99 ng/mL), and high level (100–150.99 ng/mL). The majority of patients (68.5%) were classified as vitamin D (25(OH)D3)-deficient, while 31.5% had optimal levels. No patients were found to have high vitamin D levels. This distribution indicates a high prevalence of vitamin D (25(OH)D3) deficiency in the analyzed sample, which could influence the severity and progression of oropharyngeal infections.

### 2.4. Analyzed Parameters

For each patient, the following parameters were collected and analyzed:

Demographic data: age, sex, and place of residence.

Serum vitamin D levels: categorized as deficiency (<20 ng/mL), insufficiency (20–29 ng/mL), and optimal level (≥30 ng/mL).

Infection severity: need for surgery, length of hospitalization.

Inflammatory markers: C-reactive protein (CRP), erythrocyte sedimentation rate (ESR), and leukocyte count.

### 2.5. Statistical Analysis

Data were processed using advanced statistical software (SPSS, version 20, IBM, Armonk, NY, USA) [14]. Inter-group comparisons were performed using Student’s *t*-test for continuous variables and the chi-square test, Mann–Whitney test, and ANOVA test for categorical variables. Associations between vitamin D levels and infection severity were analyzed using logistic regression models, adjusting for potential confounders. A *p*-value of <0.05 was considered statistically significant.

### 2.6. Ethical Considerations

This study received official approval from the Ethics Board of the medical institution (Approval No. 9410/08.04.2021). All patient-related data were fully anonymized to ensure privacy protection and compliance with ethical research guidelines.

## 3. Results

### 3.1. Demographic Description

The analysis of patient distribution based on vitamin D (25(OH)D3) levels and demographic characteristics did not reveal statistically significant differences (Table 1). The mean age of patients with vitamin D deficiency (25(OH)D3) was 36.91 years (±17.61), while for those with optimal levels, it was 37.09 years (±17.58). The Mann–Whitney (Z) statistical test yielded a value of −0.192, with *p* = 0.848, indicating no significant association between age and vitamin D levels.

Regarding gender distribution, 30.6% of patients with vitamin D deficiency (25(OH)D3) were women, and 38% were men. In the group with optimal levels, 12% were women, and 19.4% were men. No patients with high vitamin D (25(OH)D3) levels (≥100 ng/mL) were recorded. The statistical test did not show a significant difference (Z = −0.618, *p* = 0.537), suggesting that vitamin D (25(OH)D3) levels do not vary by gender.

In terms of geographic origin, 37% of patients with vitamin D (25(OH)D3) deficiency were from rural areas, compared to 14.8% of those with optimal levels. Among urban residents, 31.5% of patients had vitamin D deficiency, while 16.7% had optimal levels. The statistical test (Z = −0.673, *p* = 0.501) indicated no significant association between geographic origin and vitamin D levels, suggesting that factors such as sun exposure or residential characteristics do not significantly influence the distribution of vitamin D (25(OH)D3) in this sample.

Overall, the analyzed data suggest that vitamin D (25(OH)D3) levels are not significantly influenced by age, gender, or geographic origin. This finding implies that other factors, such as diet, comorbidities, or lifestyle, may have a greater impact on vitamin D (25(OH)D3) status in these patients.

### 3.2. Analysis of Patient Distribution by Vitamin D (25(OH)D3) Level and Type of Infection

The analysis of patient distribution based on vitamin D (25(OH)D3) levels and type of infection indicates a high prevalence of vitamin D (25(OH)D3) deficiency among patients with severe oropharyngeal infections (Table 2). Among these patients, 68.5% had vitamin D deficiency, while only 31.5% had optimal levels. No patient was identified with a high vitamin D (25(OH)D3) level (100–150.99 ng/mL).

In the vitamin D deficiency group, the most common infections were peritonsillar abscesses (39.8%), followed by laterocervical/submandibular abscesses (22.2%) and peritonsillar phlegmons (6.5%). This suggests that patients with vitamin D (25(OH)D3) deficiency were more prone to severe infections, particularly peritonsillar abscesses, which often required more aggressive treatment.

In contrast, patients with optimal vitamin D (25(OH)D3) levels had a significantly lower incidence of severe infections, with only 0.9% diagnosed with peritonsillar phlegmons, 9.3% with laterocervical/submandibular abscesses, and 21.3% with peritonsillar abscesses. This distribution suggests that maintaining an optimal vitamin D (25(OH)D3) level may have a protective effect against severe forms of infection.

The absence of patients with high vitamin D (25(OH)D3) levels may reflect a widespread deficiency in the study population or suggest that patients with severe oropharyngeal infections are predisposed to lower vitamin D levels. This finding further supports the hypothesis that vitamin D (25(OH)D3) may influence immune responses and the progression of oropharyngeal infections.

When categorizing surgical interventions by infection type, we observed that nearly all cases of laterocervical/submandibular abscesses (94.1%) and all cases of peritonsillar phlegmons (100%) required surgery, regardless of vitamin D status. For peritonsillar abscesses, surgery was also common, with over 80% of patients requiring an invasive procedure in both vitamin D groups. These results suggest that the type of infection plays a critical role in the decision for surgical management, independent of vitamin D status.

**Figure 1:**
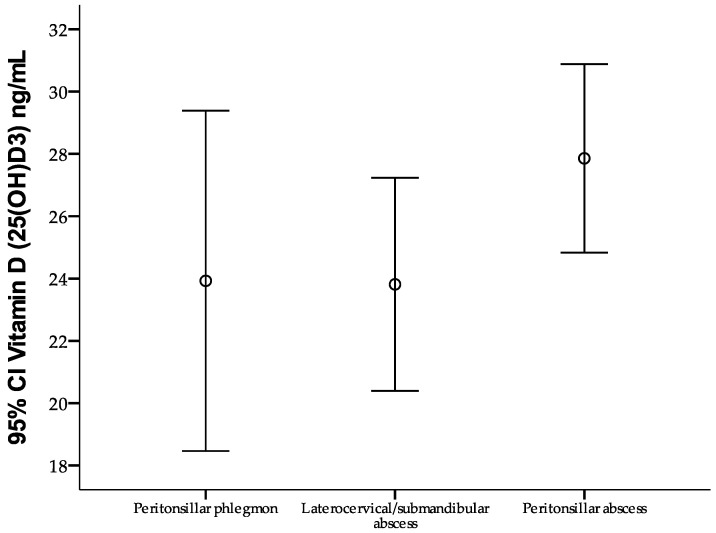
Confidence intervals (95% CIs) for vitamin D (25(OH)D3) levels by type of oropharyngeal infection, the circle representing the average vitamin D value for each oropharyngeal infection.

The results presented in Figure 1 suggest that low levels of vitamin D (25(OH)D3) are more frequently associated with severe infections, particularly peritonsillar abscesses, highlighting the need for further studies to elucidate the mechanisms through which this vitamin influences disease severity. The figure illustrates the variation in serum vitamin D levels among patients with peritonsillar phlegmons, laterocervical/submandibular abscesses, and peritonsillar abscesses. Differences between groups are evident, with a trend toward higher vitamin D levels in patients with peritonsillar abscesses compared to those with other types of infections.

### 3.3. Association Between Vitamin D Levels and Inflammatory Markers in Patients with Oropharyngeal Infections

The analysis of vitamin D levels in relation to inflammatory markers (CRP, ESR, and leukocytes) revealed significant differences in C-reactive protein (CRP) levels but not for the other parameters examined (Table 3).

Regarding CRP, patients with vitamin D deficiency had a significantly higher mean value (130.00 mg/dL, SD = 83.30) compared to those with optimal vitamin D levels (19.80 mg/dL, SD = 13.91). The ANOVA test indicated a statistically significant difference (F = 57.407, *p* = 0.001), suggesting that vitamin D deficiency is associated with a markedly higher systemic inflammatory response. This finding supports the hypothesis that vitamin D plays a crucial role in immune response regulation.

For the erythrocyte sedimentation rate (ESR), the mean values were similar between the two groups (40.3 mm/h vs. 41.6 mm/h), and the ANOVA test did not indicate a significant difference (F = 1.273, *p* = 0.239). This result suggests that, unlike CRP, the ESR is not significantly influenced by vitamin D levels, possibly due to greater variability in this inflammatory marker among patients with oropharyngeal infections.

Regarding leukocyte count, patients with vitamin D deficiency had a mean value of 14.73 (SD = 4.77), while those with optimal vitamin D levels had a slightly higher value of 15.16 (SD = 6.44). However, the difference was not statistically significant (F = 2.708, *p* = 0.103), indicating that leukocytosis is not directly influenced by vitamin D levels or that other contributing factors may affect the variability of this inflammatory marker.

Thus, only CRP showed a significant difference between the groups, suggesting that patients with vitamin D deficiency experience a more intense inflammatory response, which may contribute to the severity of the infection. In contrast, the ESR and leukocyte counts did not show significant differences, indicating that these markers are not directly influenced by vitamin D levels in oropharyngeal infections.

**Figure 2:**
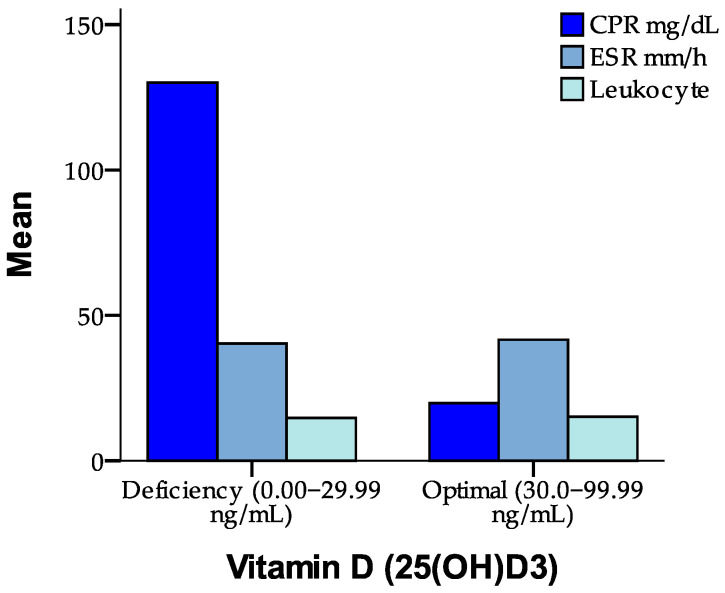
Association between vitamin D levels and inflammatory markers (CRP, ESR, and leukocytes).

These findings further support the hypothesis that vitamin D exerts an important immunomodulatory effect, particularly on systemic inflammation. Figure 2 compares the mean values of CRP (mg/dL), the ESR (mm/h), and leukocytes according to vitamin D status (deficiency vs. optimal level). The figure demonstrates that patients with vitamin D deficiency have significantly higher CRP levels, indicating a more pronounced systemic inflammatory response compared to those with optimal vitamin D levels.

### 3.4. Association Between Vitamin D Levels, Surgery, and Treatment Administered

The analysis of the relationship between vitamin D levels (25(OH)D3) and the need for surgery indicates that patients with vitamin D deficiency (25(OH)D3) (0–29.99 ng/mL) were significantly more likely to require surgical treatment compared to those with optimal levels (30–99.99 ng/mL) (Table 4). Specifically, 55.6% of patients with vitamin D deficiency required surgery, whereas only 27.8% of those with optimal levels underwent surgical intervention. Additionally, only 13% of patients with vitamin D deficiency did not require surgery, compared to 3.7% of those with optimal vitamin D levels. The chi-square test indicated a statistically significant difference (χ^2^ = 10.000, *p* = 0.002), confirming a strong association between low vitamin D (25(OH)D3) levels and the need for surgery.

Regarding the treatment administered, it was observed that patients with vitamin D (25(OH)D3) deficiency required more complex therapeutic interventions. Among them, 48.1% received a combination of antibiotics and analgesics, compared to 31.5% of patients with optimal vitamin D levels. Additionally, 10.2% of patients with deficiency required a combined regimen of antibiotics, analgesics, and antipyretics, while another 10.2% required corticosteroid therapy. In contrast, none of the patients with optimal vitamin D (25(OH)D3) levels required these additional treatments. Although the chi-square test suggested a trend toward statistical significance for antibiotic and analgesic treatment (χ^2^ = 3.767, *p* = 0.052), the difference did not reach the threshold for statistical significance.

These results suggest that vitamin D (25(OH)D3) deficiency is associated with increased severity of oropharyngeal infections, leading to a higher likelihood of surgical intervention and the need for more intensive treatments. Although the association with treatment complexity did not reach statistical significance, the observed trend indicates that patients with vitamin D (25(OH)D3) deficiency may require more therapeutic measures to control the infection effectively. These findings further support the hypothesis that vitamin D (25(OH)D3) plays a crucial role in immune response regulation and the progression of oropharyngeal infections.

### 3.5. Association Between Vitamin D (25(OH)D3) Levels and Length of Hospital Stay

The analysis of the relationship between vitamin D (25(OH)D3) levels and the length of hospital stay reveals significant differences between patients with vitamin D (25(OH)D3) deficiency (0–29.99 ng/mL) and those with optimal levels (30–99.99 ng/mL).

Patients with vitamin D (25(OH)D3) deficiency had a mean hospital stay of 8.50 days, whereas those with optimal levels had a significantly shorter duration of only 3.24 days.

The ANOVA test (F = 45.103, *p* = 0.001) indicated a statistically significant difference between the groups, suggesting that vitamin D (25(OH)D3) levels significantly influence hospitalization duration.

These results suggest that patients with vitamin D (25(OH)D3) deficiency require longer hospitalization, which may indicate a more severe course of infection and a slower recovery. This finding supports the hypothesis that vitamin D (25(OH)D3) plays a crucial role in the immune response and healing process, with its deficiency potentially contributing to a more complicated progression of oropharyngeal infections.

**Figure 3:**
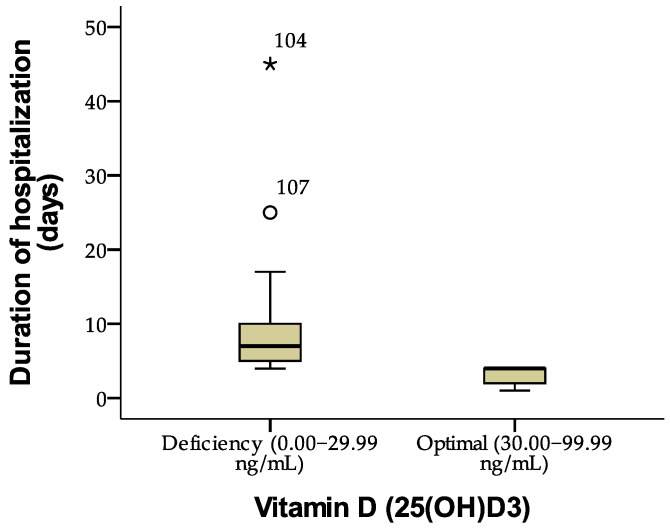
Distribution of length of hospital stay according to vitamin D level, where ° is mild outlier, and * represent extreme outlier.

Figure 3, a boxplot diagram, illustrates the differences in hospitalization length between patients with vitamin D (25(OH)D3) deficiency (0.00–29.99 ng/mL) and those with optimal levels (30.00–99.99 ng/mL). A significantly longer hospitalization duration is observed in patients with vitamin D deficiency, along with the presence of extreme values, indicating increased variability in this group.

## 4. Discussion

Our study demonstrated a significant association between vitamin D deficiency (in its precursor form) and the severity of purulent oropharyngeal infections, where vitamin D, upon being processed by the liver, is converted into 25(OH)D3, which then exerts its biological effects. Patients with low vitamin D levels experienced a more severe disease course, requiring more frequent surgical interventions and longer hospital stays.

These findings align with existing literature, which highlights the essential role of vitamin D in immune system function [8,15]. Beyond its well-established role in maintaining healthy bones and teeth, vitamin D also plays a crucial role in the proper functioning of the immune, muscular, and nervous systems [16]. Furthermore, vitamin D deficiency has been linked to disruptions in biochemical processes that contribute to type 2 diabetes, including impaired pancreatic beta-cell function, insulin resistance, and inflammation [17,18]. Prospective observational studies have shown that higher blood levels of vitamin D are associated with a lower incidence of type 2 diabetes [19].

Peritonsillar abscesses were the most frequently observed condition across both groups, likely reflecting their higher general incidence in oropharyngeal infections rather than a direct relationship with vitamin D levels. This finding is consistent with global trends where peritonsillar abscesses are among the most common deep neck infections. The high rate of surgical intervention observed in both groups may be attributed to the fact that our institution treats a referral population with more advanced infections. Indications for surgery included the presence of large abscesses, airway obstruction risk, failure of antibiotic therapy, and signs of systemic sepsis.

Additionally, vitamin D is vital for protecting the body against infectious agents, exerting immunomodulatory effects that influence the response to both viral and bacterial infections [20]. Its deficiency may be a vulnerability factor, particularly in at-risk populations such as children, the elderly, and individuals with comorbidities [21,22,23]. Given the current epidemiological landscape, where the spread of infectious agents remains unpredictable, optimizing vitamin D levels could serve as an effective strategy to mitigate the severity of infectious diseases.

In our study, we did not observe a significant correlation between geographic origin or other demographic factors such as age, sex, or vitamin D levels. One potential explanation for this finding is limited ultraviolet radiation (UVR) exposure, particularly UVB, which is essential for the conversion of 7-dehydrocholesterol (7DHC) to vitamin D3 in the skin [24]. This process is highly dependent on adequate UVB exposure, and individuals with limited sun exposure, whether due to geographic location, lifestyle, or other factors, may exhibit lower levels of vitamin D synthesis despite residing in areas where vitamin D deficiency is common. While our study did not assess UVR exposure directly, future studies could benefit from incorporating data on patients’ sun exposure to better understand how UVB levels contribute to vitamin D status and, consequently, infection severity.

It is also important to note that vitamin D3 can be metabolized via an alternative pathway, initiated by CYP11A1, leading to the production of biologically active hydroxyderivatives independent of 25(OH)D3 [25]. These metabolites have been reported to exert significant immunomodulatory and anti-inflammatory effects and may contribute to local immune regulation at the site of infection, including the oropharyngeal mucosa. Although our study focused on serum 25(OH)D3 levels, this additional pathway highlights that vitamin D’s biological impact extends beyond the classical endocrine actions. Further research considering these alternative metabolites could offer a more comprehensive understanding of vitamin D’s role in infection severity and immune response [26].

It is also important to recognize that various vitamin D compounds, including different metabolites and precursors, are present in a wide range of nutritional products. These compounds, which are distinct from the commonly recognized vitamin D3 used in food fortification, may also play a role in supporting adequate vitamin D status. For example, some nutritional supplements may contain forms such as vitamin D2 (ergocalciferol) or other bioactive derivatives, each contributing differently to the body’s vitamin D pool. This is particularly relevant when considering the variability in vitamin D status across different populations, as some may benefit from alternative vitamin D compounds present in their diet. Unlike food fortification, which often focuses on adding vitamin D3 to a limited range of foods, the broader spectrum of vitamin D metabolites in nutritional products can influence vitamin D metabolism and immune system function in ways not captured by fortification efforts alone [27].

Our study demonstrates that vitamin D levels significantly influence the inflammatory response, particularly in relation to C-reactive protein (CRP). Patients with vitamin D deficiency exhibited significantly higher CRP values compared to those with optimal vitamin D levels (130.00 mg/dL vs. 19.80 mg/dL, *p* = 0.001), indicating a pronounced systemic inflammatory response. This finding is consistent with previous studies emphasizing the immunomodulatory and anti-inflammatory properties of vitamin D. A possible explanation is that vitamin D modulates macrophage activity and proinflammatory cytokine production, thereby reducing excessive inflammatory responses to severe infections.

In contrast, the erythrocyte sedimentation rate (ESR) and leukocyte count did not show significant differences between the vitamin D deficiency and optimal-level groups (*p* = 0.239 and *p* = 0.103, respectively). This suggests that these markers are not influenced to the same extent by vitamin D status. The results indicate that vitamin D has a more pronounced effect on acute inflammation (as measured by CRP) rather than on chronic inflammatory markers like the ESR. Additionally, the variability in the ESR and leukocyte count may be influenced by other factors such as infection severity, nutritional status, or comorbidities [28,29,30,31,32,33,34]. While our study found significantly higher C-reactive protein (CRP) levels in patients with vitamin D deficiency, the erythrocyte sedimentation rate (ESR) and leukocyte counts did not show significant differences between the groups. This inconsistency may be attributed to greater variability in these markers or their influence by additional factors such as comorbidities, infection severity, or individual immune responses. We recommend that future studies explore additional inflammatory markers or perform longitudinal measurements to gain a better understanding of how vitamin D levels affect inflammation during infection.

These findings reinforce the importance of maintaining adequate vitamin D levels in controlling inflammation [35,36,37,38,39]. They also suggest that vitamin D supplementation, which is metabolized into 25(OH)D3 in the liver, may help improve the outcomes of these infections [40,41,42,43,44,45].

### 4.1. Clinical Implications and Future Directions

Our results, along with existing evidence, suggest that maintaining sufficient vitamin D (25(OH)D3) levels could positively impact the progression of purulent oropharyngeal infections and other conditions associated with inflammation and immune dysfunction. These findings highlight the need for active screening of vitamin D levels in patients with severe infections and the integration of preventive measures such as supplementation or dietary modifications. We agree that clinical trials on vitamin D supplementation for oropharyngeal infections would be valuable. Future randomized controlled trials should explore its potential to reduce infection severity in vitamin D-deficient patients.

### 4.2. Study Limitations

This study has several limitations that should be acknowledged:

Retrospective Design: The retrospective nature of the study limits the ability to establish causality between vitamin D levels and infection severity. Data collection from medical records may also introduce recording errors or uncontrolled confounding factors. Due to the retrospective design, our study could not account for fluctuations in vitamin D levels. Longitudinal studies with repeated measurements would provide better insight into how changes in vitamin D status affect infection outcomes.

Unaccounted Variables: Factors such as sun exposure, nutritional status, and prior vitamin D supplementation were not included in the analysis, which may have influenced the results.

Unequal Distribution of Vitamin D Levels: No patients with high vitamin D levels (>100 ng/mL) were identified, restricting the ability to assess the potential protective effect of elevated vitamin D (25(OH)D3) levels on infection severity.

We acknowledge that several potential confounders, such as sun exposure, dietary habits, and prior vitamin D supplementation, may influence both vitamin D levels and infection severity. Unfortunately, due to the retrospective nature of the study and limitations in available data, we could not account for these factors. Future studies should incorporate data on lifestyle factors and previous vitamin D supplementation to control for these confounders and provide more accurate conclusions regarding the role of vitamin D in infection severity.

Sample Size and Generalizability: Studying a larger, more diverse sample with a prospective design could improve the generalizability of the findings.

### 4.3. Future Research Perspectives

Future research should focus on the following:

Assessing the impact of vitamin D (25(OH)D3) supplementation on the progression and severity of oropharyngeal infections.

Investigating the underlying immunological mechanisms by which vitamin D deficiency influences the immune response in severe infections.

Conducting multicenter studies with large cohorts and long-term follow-up to determine whether optimizing vitamin D (25(OH)D3) levels can be effectively integrated as a preventive or therapeutic strategy for severe upper respiratory tract infections.

## 5. Conclusions

The study revealed a significant association between vitamin D deficiency (25(OH)D3) and the severity of purulent oropharyngeal infections, supporting the hypothesis that low vitamin D (25(OH)D3) levels contribute to a more severe disease course.

Patients with vitamin D (25(OH)D3) deficiency had a significantly longer hospital stay (8.50 days vs. 3.24 days, *p* = 0.001), suggesting a slower response to treatment and a more challenging recovery. These patients also required surgical interventions more frequently (55.6% vs. 27.8%, *p* = 0.002), indicating a predisposition to more aggressive forms of infection.

Regarding treatment, patients with low vitamin D (25(OH)D3) levels showed an increased tendency to require more complex therapeutic approaches, including corticosteroid therapy and combined antibiotic-antipyretic treatments, although this association was not statistically significant (*p* > 0.05).

Vitamin D deficiency was also linked to a more intense systemic inflammatory response, as reflected by significantly elevated C-reactive protein (CRP) levels, which may contribute to the severity of oropharyngeal infections.

These findings underscore the importance of assessing and optimizing vitamin D (25(OH)D3) levels in patients with severe infections to improve prognosis and reduce the need for aggressive therapeutic interventions. Future studies should explore the impact of vitamin D (25(OH)D3) supplementation on the course of oropharyngeal infections to determine whether it may have a preventive or therapeutic role.

## Figures and Tables

**Table 1:** Distribution of patients according to vitamin D level (25(OH)D3).

Parameters		Vitamin D Level (25(OH)D3)				Z	*p*
		Deficiency (0.00–29.99 ng/mL)		Optimal (30.00–99.99 ng/mL)			
		N	%	N	%		
Age (Mean ± SD)		36.91 ± 17.61		37.09 ± 17.58		−0.192	0.848
Sex	Female	33	30.6	13	12.0	−0.618	0.537
	Male	41	38.0	21	19.4		
Environment	Rural	40	37.0	16	14.8	−0.673	0.501
	Urban	34	31.5	18	16.7		

N = number of patients, SD = standard deviation, *p* = statistical significance, Z = Mann–Whitney coefficient.

**Table 2:** Distribution of patients according to vitamin D (25(OH)D3) category and type of infection.

Parameters	Vitamin D Level (25(OH)D3)						χ^2^	*p*
	Deficiency (0.00–29.99 ng/mL)		Optimal (30.00–99.99 ng/mL)		Total			
	N	%	N	%	N	%		
Peritonsillar phlegmons	Yes	36	83.7	19	55	83.3	4.500	0.034
	No	7	16.3	4	11	16.7		
Laterocervical/submandibular abscesses	Yes	23	95.8	9	32	94.1	5.765	0.016
	No	1	4.2	1	2	5.9		
Peritonsillar abscesses	Yes	7	100	1	8	100	6.061	0.014
	No	0	0	0	0	0		

N = number of patients, *p* = statistical significance, **χ**^2^ = chi-square coefficient.

**Table 3:** Association between vitamin D Levels and inflammatory markers (CRP, ESR, and leukocytes) in patients with oropharyngeal infections.

Parameters	Vitamin D Level (25(OH)D3)				F	*p*
	Deficiency (0.00–29.99 ng/mL)		Optimal (30.00–99.99 ng/mL)			
	Mean	SD	Mean	SD		
CPR mg/dL	130.00	83.30	19.80	13.91	57.407	0.001
ESR mm/h	40.3	25.6	41.6	29.4	1.273	0.239
Leukocytes	14.73	4.77	15.16	6.44	2.708	0.103

SD = standard deviation, *p* = statistical significance, F = ANOVA coefficient, CPR = C-reactive protein, ESR = erythrocyte sedimentation rate.

**Table 4:** Association between vitamin D levels, surgery, and treatment administered.

Parameters		Vitamin D Level (25(OH)D3)				χ^2^	*p*
		Deficiency (0.00–29.99 ng/mL)		Optimal (30.00–99.99 ng/mL)			
		N	%	N	%		
Surgery	No	14	13.0	4	3.7	5.556	0.018
	Yes	60	55.6	30	27.8	10.000	0.002
Treatment	Antibiotics, analgesics	52	48.1	34	31.5	3.767	0.052
	Antibiotics, analgesics, antipyretics	11	10.2	0	0.0	-	-
	Antibiotics, analgesics, antipyretics corticosteroid therapy	11	10.2	0	0.0	-	-

N = number of patients, *p* = statistical significance, χ^2^ = chi-square coefficient.

## Data Availability

All the data processed in this article are part of the research for a doctoral thesis and are archived in the aesthetic medical office, where the interventions were performed.
